# Characteristics and innovative points of clinical trials of radiotherapy combined with immune checkpoint inhibitors in NSCLC over the past decade

**DOI:** 10.3389/fmed.2025.1598505

**Published:** 2025-07-22

**Authors:** Mengting Li, Peng Ding

**Affiliations:** ^1^Cancer Center, Union Hospital, Tongji Medical College, Huazhong University of Science and Technology, Wuhan, China; ^2^Tongji Medical College, Institute of Radiation Oncology, Union Hospital, Huazhong University of Science and Technology, Wuhan, China; ^3^Hubei Key Laboratory of Precision Radiation Oncology, Wuhan, China

**Keywords:** non-small cell lung cancer, immune checkpoint inhibitors, radiotherapy, clinical trials, immunoradiotherapy

## Abstract

**Background:**

This study aims to statistically and qualitatively evaluate the characteristics of immunoradiotherapy (iRT) clinical trials for non-small cell lung cancer (NSCLC) registered on the ClinicalTrials.gov website over the past decade, to help researchers grasp current research trends and design higher-quality iRT clinical trials in the future.

**Methods:**

We conducted a cross-sectional, descriptive study of interventional non-small cell lung cancer iRT clinical trials registered from 2014 to 2024. This study focuses on the combination of different radiotherapy methods with immune checkpoint inhibitors (ICIs), minimizing attention to new immunotherapeutic drugs. It emphasizes the exploration of radiotherapy and suitable patient populations. Therefore, the types of ICIs are limited to PD-1/PD-L1 and CTLA-4 inhibitors, and the main innovative points of the included clinical trials were categorized and statistically analyzed.

**Results:**

As of 24 June 2024, 196 clinical trials were available for analysis. Among these trials, more than 76% of clinical trials focused on patients with stage III and higher NSCLC. About 35.2% of the studies were still recruiting, only 14.8% were marked as completed, and 12.8% had failed, with slow enrollment, safety, and funding issues being the main reasons for failure. Phase 2 trials (56.1%) led significantly, with only 11.7% of trials reaching phase 3; hence, 55.6% had a sample size of fewer than 50 participants. Nearly half (45.4%) of the studies were multi-center trials, and 54.6% had data monitoring. Durvalumab was explicitly mentioned in 30.1% of the studies. Most clinical trials (64.3%) focused on innovating radiotherapy dose adjustments, with 104 studies adopting a hypofractionated radiotherapy-based protocol.

**Conclusion:**

The number of iRT clinical trials in the NSCLC field is rapidly increasing. Most patients are in locally advanced stages or higher, with phase 2 trials predominating. Durvalumab is the representative drug, and researchers are particularly interested in optimizing radiotherapy doses, with a tendency to adopt hypofractionated radiotherapy.

## Background

Immunoradiotherapy (iRT) has aroused great attention in the field of non-small cell lung cancer (NSCLC) as an innovative cancer treatment strategy over the past decade (1). Radiotherapy can not only damage tumor cells’ DNA duplexes directly and indirectly, leading to apoptosis or necrosis, but also induce immunogenic death (ICD) of tumor cells, which releases tumor-associated antigens (TAAs), thereby activating the host immune system and relieving immunosuppression in the tumor microenvironment, enhancing the recognition and clearance of tumors by immune cells, and avoiding immune escape of tumor cells (2). This immunostimulatory effect provides a theoretical basis for the combination of radiotherapy and immunotherapy, especially with immune checkpoint inhibitors (ICIs), to achieve the purpose of “local treatment promoting systemic immune response.”

Only three clinical trials started to try this combined modality therapy in 2014, and the PACIFIC study was one of them (3). Its findings have aroused much concern after publication, and since then the number of iRT clinical trials has surged globally, with 25 iRT-related clinical trials initiated in 2017 alone, establishing the PD-L1 inhibitor durvalumab as a representative drug for such trials. Innovations in clinical trials are to find appropriate populations and innovate interventions. The patient populations include, but are not limited to, those with unresectable locally advanced, oligometastatic, brain metastatic, and even early-stage lung cancer. iRT is not a simple superposition of the two treatment modalities, and it is necessary to take into account the selection of ICIs and radiotherapy techniques, and the adjustment of irradiation dose. From conventional radiotherapy to proton and heavy ion therapy, stereotactic radiosurgery (SRS) for brain metastases to low-dose radiation therapy (LDRT), adaptive radiotherapy, dose-painting, and various ICIs came out one after another to participate in the trial.

Clinical trials are pivotal in assessing the efficacy of new drugs for specific diseases, playing a crucial role in driving medical innovation and developing more effective treatments (4). Therefore, a thorough analysis of registered clinical trial data is invaluable for shaping future clinical practice standards (5). ClinicalTrials.gov is a public trial registration center provided by the U. S. National Library of Medicine and the U. S. Food and Drug Administration and accounts for more than 80% of all studies on the World Health Organization International Clinical Trials Registry Platform (6). Consequently, we designed and conducted a cross-sectional study to systematically review and analyze clinical trial information on iRT in the field of NSCLC registered on this platform. This study aims to have a more comprehensive understanding of the characteristics, concerns, and development trends of iRT clinical trials in the NSCLC field over the last decade, provide data support from a different perspective for future research innovations and therapeutic advancements, anticipating the emergence of more high-quality iRT clinical trials.

## Methods

We conducted a cross-sectional, descriptive study on interventional non-small cell lung cancer iRT clinical trials registered from 2014 to 2024. Considering that the combination of some new immunotherapeutic drugs with radiotherapy is not the main purpose of the study, but lies in testing the safety of new drugs, this study will focus on the combination of different radiotherapy methods with ICIs, reduce the attention to new immunotherapeutic drugs, and highlight the exploration of radiotherapy and appropriate population. Therefore, the types of ICIs are limited to PD-1/PD-L1 and CTLA-4 inhibitors, and the main innovative points of the included clinical trials were categorized and statistically analyzed.

### Search term and exclusion criteria

We used the advanced search function on ClinicalTrials.gov. We searched for “NSCLC” in “Condition/disease” and “radiation and immunotherapy” in “Other terms,” and various words were also tried to replace the word “immunotherapy” to avoid omission. The specific search term can be viewed in Supplementary Table S1, and “Study Type” was selected as “Interventional.” All clinical trial records collected were exported and assessed. The following data fields were extracted: NCT number, study title, study status, study results, conditions, interventions, primary outcome measures, sponsor, collaborators, sex, age, phases, enrollment, funder type, study type, study design, start date, and locations. The data fields were imported into Excel (Microsoft Office Excel 2021, Microsoft Corporation) for further screening. Exclusion criteria included

1. Trials without specific NCT numbers or with duplicate entries.
2. Trials before 2014.
3. Trials in which the disease type is not NSCLC.
4. Trials in which the investigational drugs do not include PD-1/PD-L1 and CTLA-4 inhibitors.
5. Trials without combined radiotherapy or immunotherapy.
6. Trials combining other therapies with iRT, such as thermotherapy or tumor treating fields (TTF).
7. Trials whose primary purpose is not to explore iRT treatment modalities or efficacy, such as those observing the quality of life or biomarkers from liquid biopsies.

All trials were further subcategorized based on their data fields and innovative points. Descriptive statistics were used to characterize trial features, providing frequencies and percentages for categorical data.

### Data analysis

A descriptive analysis was conducted. In addition to categorizing data fields by the website, we further classified the sponsors into universities, hospitals, companies, foundations, research institutions, and oncology cooperative groups. These were then divided by region into North America, South America, Asia, Europe, and Oceania. Also, we categorized innovative points of all studies included, with ICIs restricted to PD-1/PD-L1 and CTLA-4 inhibitors. For radiotherapy, the innovative points were divided into four major categories: technology, dose, radiation field, and auxiliary systems. Regarding intervention population and tumor conditions, the innovative points were categorized into four major groups: patient status, primary tumor site, metastatic site, and lymph nodes. Further detailed categorization of these innovative points was based on actual occurrences. For instance, the innovative points for radiotherapy dose distribution were divided into six subcategories: hypofractionated, hyperfractionated, single fraction, low dose, hybrid dose-fraction, and other approaches to dose adjustment. No further subcategorization was made beyond this. Each study was assigned a value of 1 for the occurrence of an innovative point, which was recorded in the corresponding major and minor categories. These values could be cumulatively summed, and the number of trials in each category was statistically calculated for different years. Categorical data were reported as frequencies and percentages, while continuous variables were presented as medians and interquartile ranges.

## Results

### Trial screening

**Figure 1:**
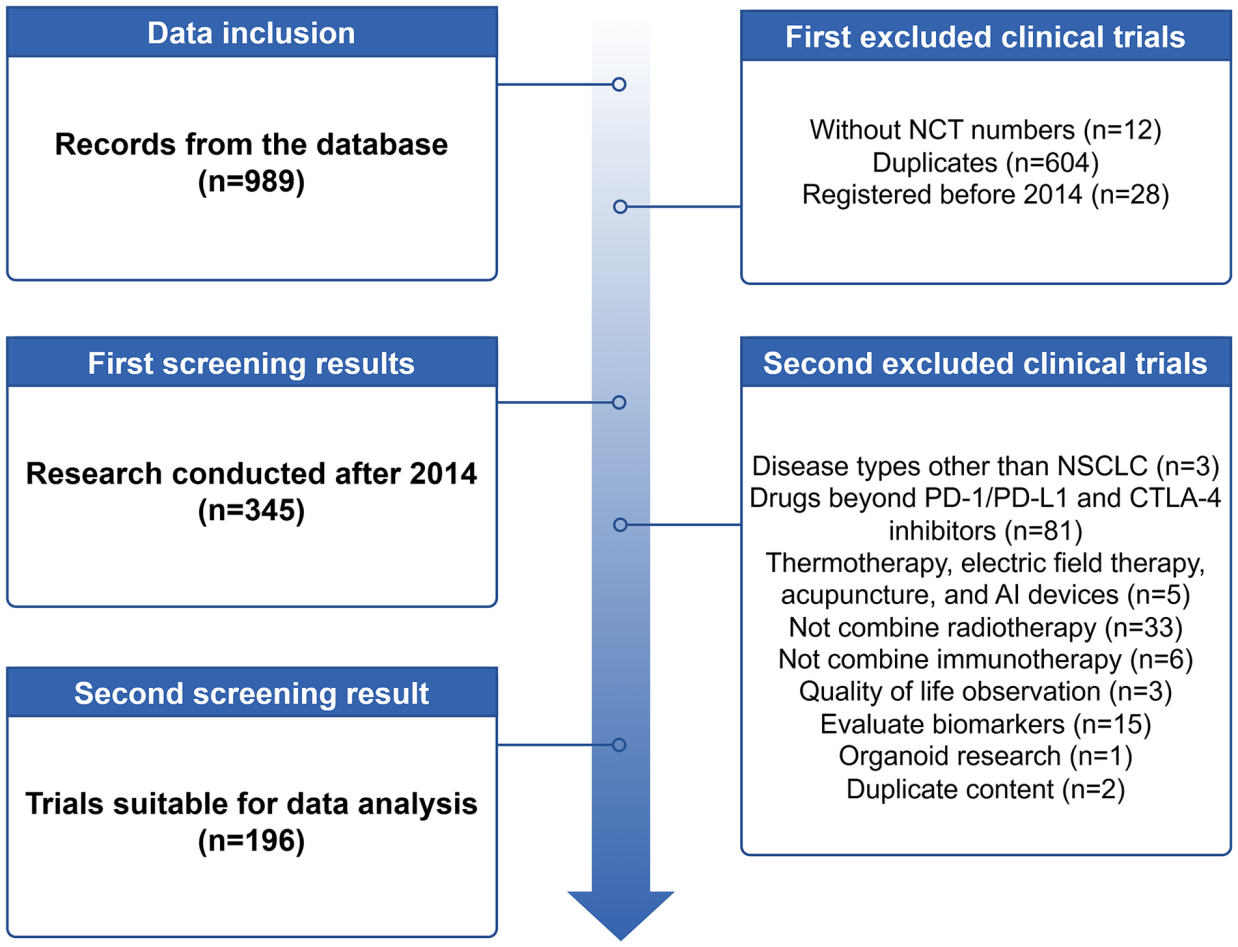
Screening flowchart for iRT clinical trials registered on the ClinicalTrial.gov from 2014 to 2024. By Figdraw.

As of 24 June, 2024, we initially searched the website for 989 registered clinical trials, of which there were many studies with duplicate NCT numbers. After excluding trials without NCT numbers and duplicates, 373 trials remained. Further exclusion of trials registered before 2014 left 345 trials. Upon careful review of all study design information, we found that 3 trials involved disease types other than NSCLC, 81 trials involved drugs beyond PD-1/PD-L1 and CTLA-4 inhibitors, 5 trials used treatment modalities such as thermotherapy, electric field therapy, acupuncture, and AI devices, 33 trials did not combine radiotherapy, 6 trials did not combine immunotherapy, 3 trials focused on quality of life observation, 15 trials aimed to evaluate biomarkers, 1 trial was related to organoid research, and 2 trials, despite having different NCT numbers, had duplicate content. Ultimately, a total of 196 interventional registered trials were included. The detailed screening process is shown in Figure 1.

### General characteristics of included studies

There were 44 trials (22.4%) between 2014 and 2017, 62 trials (31.6%) between 2018 and 2020, and rising to 90 trials (45.9%) between 2021 and 2024. 98.5% of the trials clustered in “adult, older adult.” 39.8% of trials were stage IV NSCLC, followed by 31.1% of trials with stage III NSCLC, and then some trials involved stage I-II NSCLC. In addition, 19 (9.7%) trials focused on oligometastatic, oligoprogressive, or oligoresidual populations, and 12 (6.1%) trials focused on brain or leptomeningeal metastases. Organizers were mainly hospitals (47.4%) and universities (28.1%), followed by research institutions (9.2%), companies (7.1%), oncology collaborating organizations (5.1%), and foundations (3.1%). 85.2% of the investigators labeled the funding source Other, 8.2% of the trials came from industry, 3.1% came from NIH, and 1.5 and 1% came from NETWORK and FED. About half of the trials were conducted in North America (48.5%), followed closely by Asia (29.6%) and Europe (20.4%), and iRT trials in Oceania and South America were less registered on this website. 35.2% of trials were recruiting, 19.4% were active but not recruiting, 29 trials had been completed (14.8%), 7.7% had not yet enrolled, followed by trial failure (7.7% terminated, 4.6% withdrawn, and 0.5% suspended) and unknown status (10.2%). Most investigators (89.8%) did not register study results on the website, and only 10.2% of trials were improving their study results registration. Eleven trials failed because of slow enrollment, four because of safety reasons, three because of funding issues, two because of study strategy adjustments by the sponsor, and the remaining five were due to protocol design issues, difficulty in balancing work and research, study delay and competitive enrollment, another treatment modality found to be effective, and poor early results, respectively. Characteristics of the included trials are presented in Table 1.

**Table 1:** Characteristics of iRT clinical trials for NSCLC registered on ClinicalTrials.gov.

Characteristic	*N* (%), *n* = 196
Year	
2014–2017	44 (22.4)
2018–2020	62 (31.6)
2021–2024	90 (45.9)
Age	
Adult, older adult	193 (98.5)
Child, adult, older adult	2 (1.0)
Older adult	1 (0.5)
Tumor stage	
I	6 (3.1)
I–II	8 (4.1)
I–III	7 (3.6)
II–III	25 (12.8)
II–IV	1 (0.5)
III	61 (31.1)
Oligoprogressive	2 (1.0)
III-IV	10 (5.1)
Oligometastatic, oligoresidual	1 (0.5)
IV	78 (39.8)
Oligometastatic, oligoprogressive	16 (8.2)
Brain or leptomeningeal metastasis	12 (6.1)
Sponsor	
Hospital	93 (47.4)
University	55 (28.1)
Research institutions	18 (9.2)
Company	14 (7.1)
Oncology cooperation organization	10 (5.1)
Foundation	6 (3.1)
Funder type	
Industry	16 (8.2)
NIH	6 (3.1)
Network	3 (1.5)
FED	2 (1.0)
Other GOV	2 (1.0)
Other	167 (85.2)
Locations	
North America	95 (48.5)
Asia	58 (29.6)
Europe	40 (20.4)
Oceania	2 (1.0)
South America	1 (0.5)
Study status	
Recruiting	69 (35.2)
Active not recruiting	38 (19.4)
Completed	29 (14.8)
Unknown	20 (10.2)
Not yet recruiting	15 (7.7)
Terminated	15 (7.7)
Withdrawn	9 (4.6)
Suspended	1 (0.5)
Study results	
Yes	20 (10.2)
No	176 (89.8)
Reasons for trial failure	
Due to slow accrual	11 (44.0)
Safety	4 (16.0)
Lack of funding	3 (12.0)
Sponsor strategic or business decision	2 (8.0)
Pending a protocol amendment	1 (4.0)
Work commitments	1 (4.0)
Due to the delay in the study scheduled, due to the start of competitive study	1 (4.0)
Another treatment found efficacious	1 (4.0)
Clinical decision based on previous results	1 (4.0)

### Study design included in the trial

All studies were interventional in nature, and there was no requirement for patient sex. The clinical trials were mainly staged at phase 2 (56.1%), followed by phase 3 (11.7%), phase 1 (11.2%), and phase 1–2 (8.7%). Single-center (54.6%) and multi-center (45.4%) trials accounted for approximately half each. 109 (55.6%) trials had sample sizes below 50, 41 (20.9%) trials below 100, 27 (13.8%) trials below 200, 15 (7.7%) trials below 500, and 4 (2.0%) trials above 500. The primary objective of the trial was treatment in 96.43%. Randomization was adopted in 36.7% of studies, non-randomization in 14.8% of studies, and the allocation pattern was unclear in 48.5% of studies. 93.4% of the trials adopted the open-label model, but a small number of trials adopted a blind design from single-blind to four-blind. 107 (54.6%) trials had a Data Monitoring Committee, and 89 (45.4%) did not. Detailed data are presented in Table 2.

**Table 2:** Study design element of iRT clinical trials for NSCLC.

Characteristic	*N* (%), *n* = 196
Study type	
Interventional	196 (100)
Phase	
Early phase 1	4 (2.0)
Phase 1	22 (11.2)
Phase1| Phase 2	17 (8.7)
Phase 2	110 (56.1)
Phase 2| Phase 3	2 (1.0)
Phase 3	23 (11.7)
Phase 4	1 (0.5)
NA	17 (8.7)
Single center or multi center	
Single	107 (54.6)
Multi	89 (45.4)
Enrollment	
<50	109 (55.6)
≥50, <100	41 (20.9)
≥100, <200	27 (13.8)
≥200, <500	15 (7.7)
≥500	4 (2.0)
Primary purpose	
Treatment	189 (96.4)
Supportive care	2 (1.0)
Prevention	2 (1.0)
Diagnostic	1 (0.5)
Basic science	1 (0.5)
Other	1 (0.5)
Allocation	
Randomized	72 (36.7)
Non randomized	29 (14.8)
NA	95 (48.5)
Interventional model	
Single group	101 (51.5)
Parallel	82 (41.8)
Sequential	12 (6.1)
Crossover	1 (0.5)
Masking	
None (open label)	183 (93.4)
Single (investigator)	1 (0.5)
Single (investigator)	1 (0.5)
Double (participant, care provider)	1 (0.5)
Double (participant, investigator)	4 (2.0)
Triple (participant, care provider, investigator)	1 (0.5)
Quadruple (participant, care provider, investigator, outcomes assessor)	5 (2.6)
Data monitoring committee	
Yes	107 (54.6)
No	89 (45.4)

### Optimization of treatment modalities

**Figure 2:**
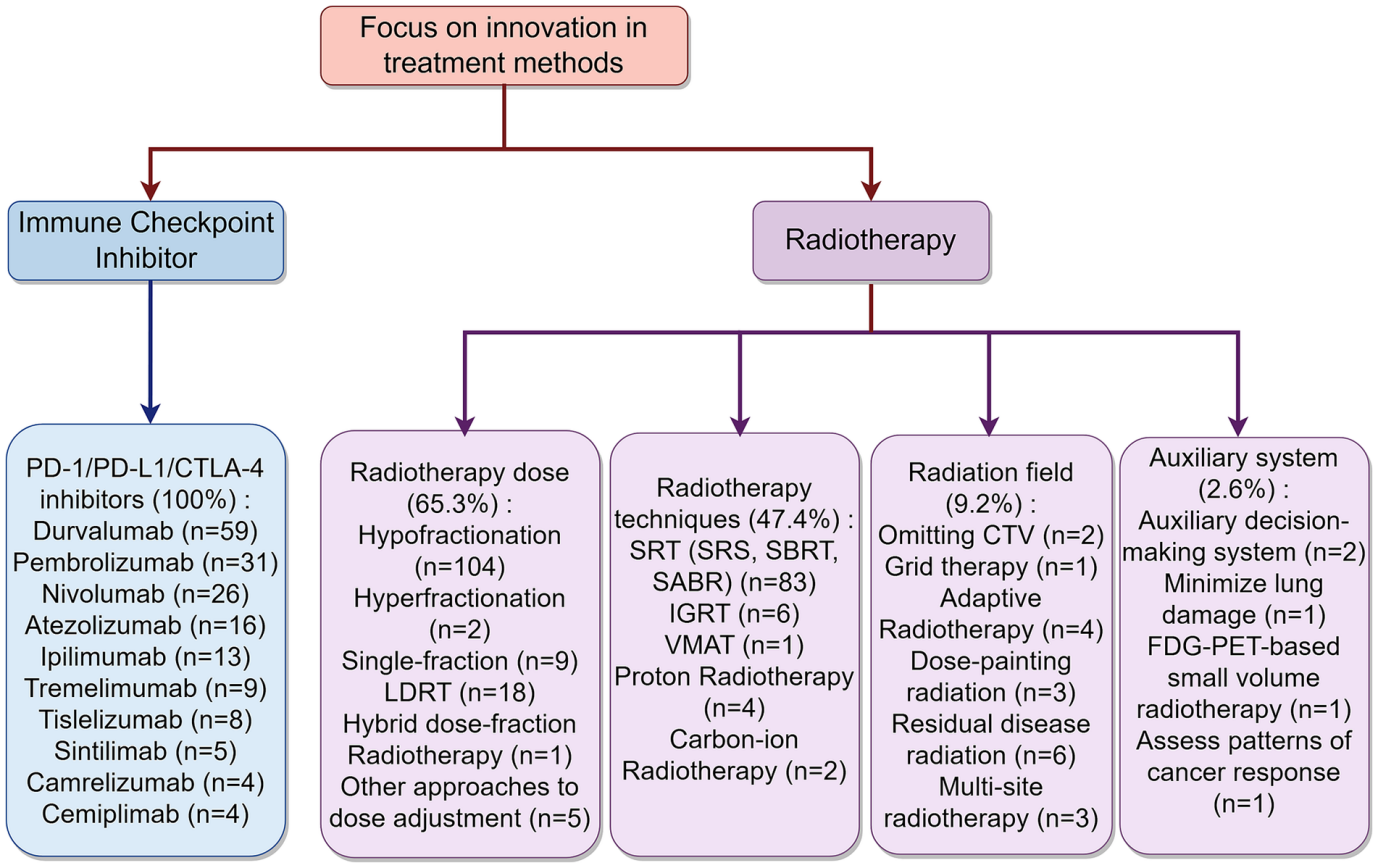
Distribution of innovative points on treatment methods. By Figdraw.

The clinical study design of iRT focused on innovations in intervention populations and intervention methods (immunotherapy and radiotherapy), categorized into 8 major categories and 36 subcategories (detailed statistical data can be found in Supplementary Table S2). Regarding ICIs, 59 studies (30.9%) explicitly mentioned durvalumab, 31 studies (15.8%) mentioned pembrolizumab, 26 studies (13.3%) mentioned nivolumab, while atezolizumab and ipilimumab were mentioned in 16 studies (8.4%) and 13 studies (6.8%), respectively. For optimization of radiotherapy modalities, more than half (65.3%) of the studies chose to innovate dose fractionation, 104 trials used hypofractionated radiotherapy, 18 studies used low-dose radiotherapy, which included combination therapy with hypofractionation, and 8 studies used a single fraction. Because different segmentations require advances in radiotherapy instrumentation, there will be overlapping parts between the two. Nearly half (47.4%) of the trials focused on innovations in radiotherapy technology, with 83 studies using stereotactic radiotherapy (SRT) (including SRS, SBRT, and SABR), 6 studies using image-guided radiotherapy (IGRT), and another 6 studies employing proton and heavy ion therapy. Regarding radiation field, 9.2% of the trials were concerned with the radiation area. Specifically, 6 studies addressed irradiating residual lesions, 4 studies implemented adaptive radiotherapy, 3 studies used dose-painting techniques, and 2 studies mentioned omitting the clinical target volume (CTV). Additionally, 2.6% focused on auxiliary radiotherapy systems, with 2 studies involving auxiliary decision-making systems and 1 study aiming to reduce lung function damage caused by iRT. A summary of the study design’s innovative points can be seen in Figure 2.

### Identifying appropriate population

**Figure 3:**
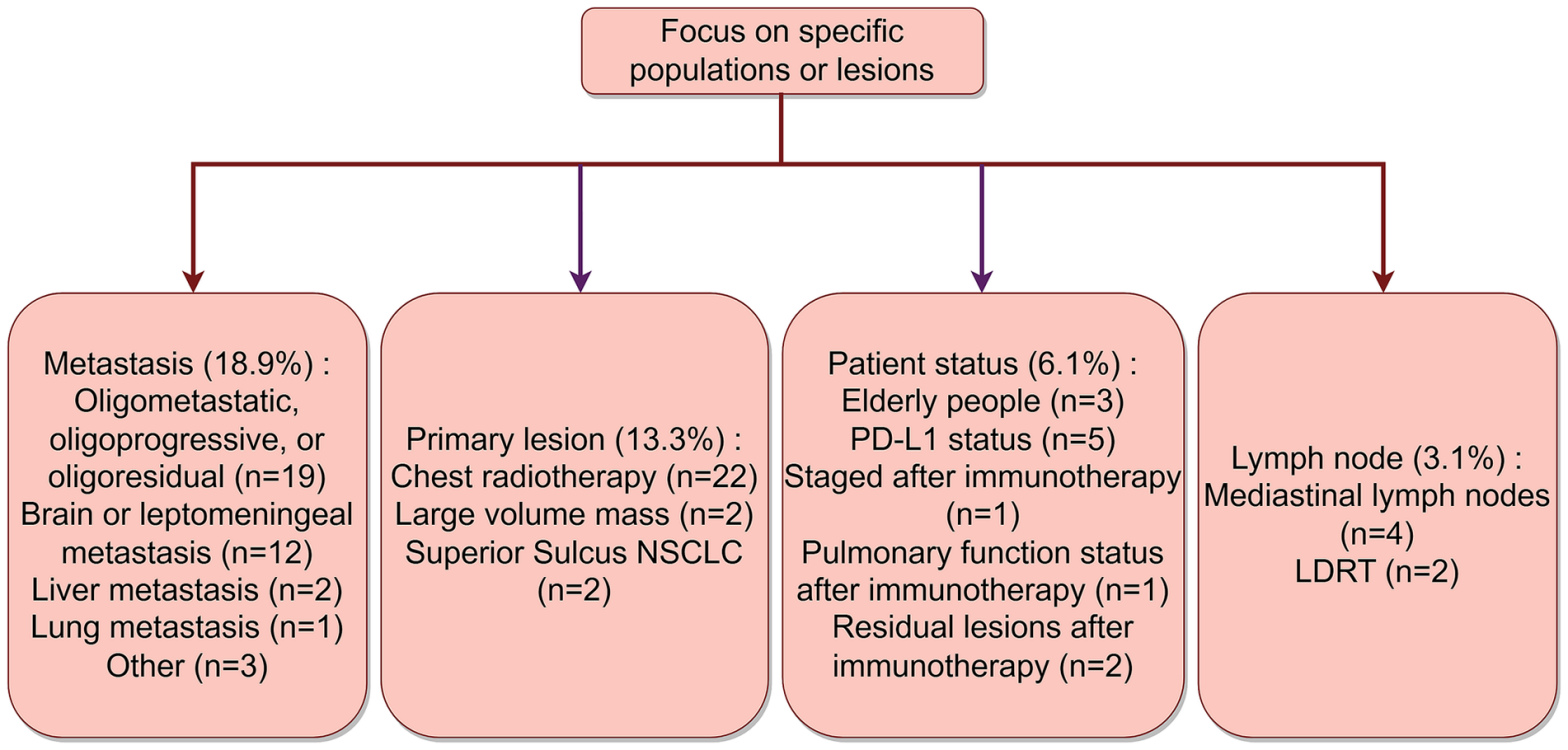
Distribution of innovative points for specific populations or lesions. By Figdraw.

Over 76% of the iRT patients were NSCLC patients with locally advanced or higher stages. Innovations targeting metastatic sites were noted in 18.9% of the studies, with 19 trials focusing on oligometastatic, oligoprogressive, or oligoresidual, 12 studies on brain or leptomeningeal metastases, and 2 studies each on liver and lung metastases. For innovations targeting primary tumor sites, 13.3% of the trials were involved. Among these, 22 trials focused on thoracic radiotherapy, 2 studies on large-volume tumors, and 2 studies on superior sulcus tumors. Additionally, 3.1% of the trials explicitly mentioned irradiation plans for lymph nodes. Furthermore, 6.1% of the trials focused on the status of the patients (including post-immunotherapy status). Five studies focused on PD-L1 status, three studies on elderly patients, two studies guided radiotherapy based on residual lesions post-immunotherapy, one based on lung function status post-immunotherapy, and one based on tumor staging post-immunotherapy. A summary of these study focuses is shown in Figure 3.

### Trends in innovative points

**Figure 4:**
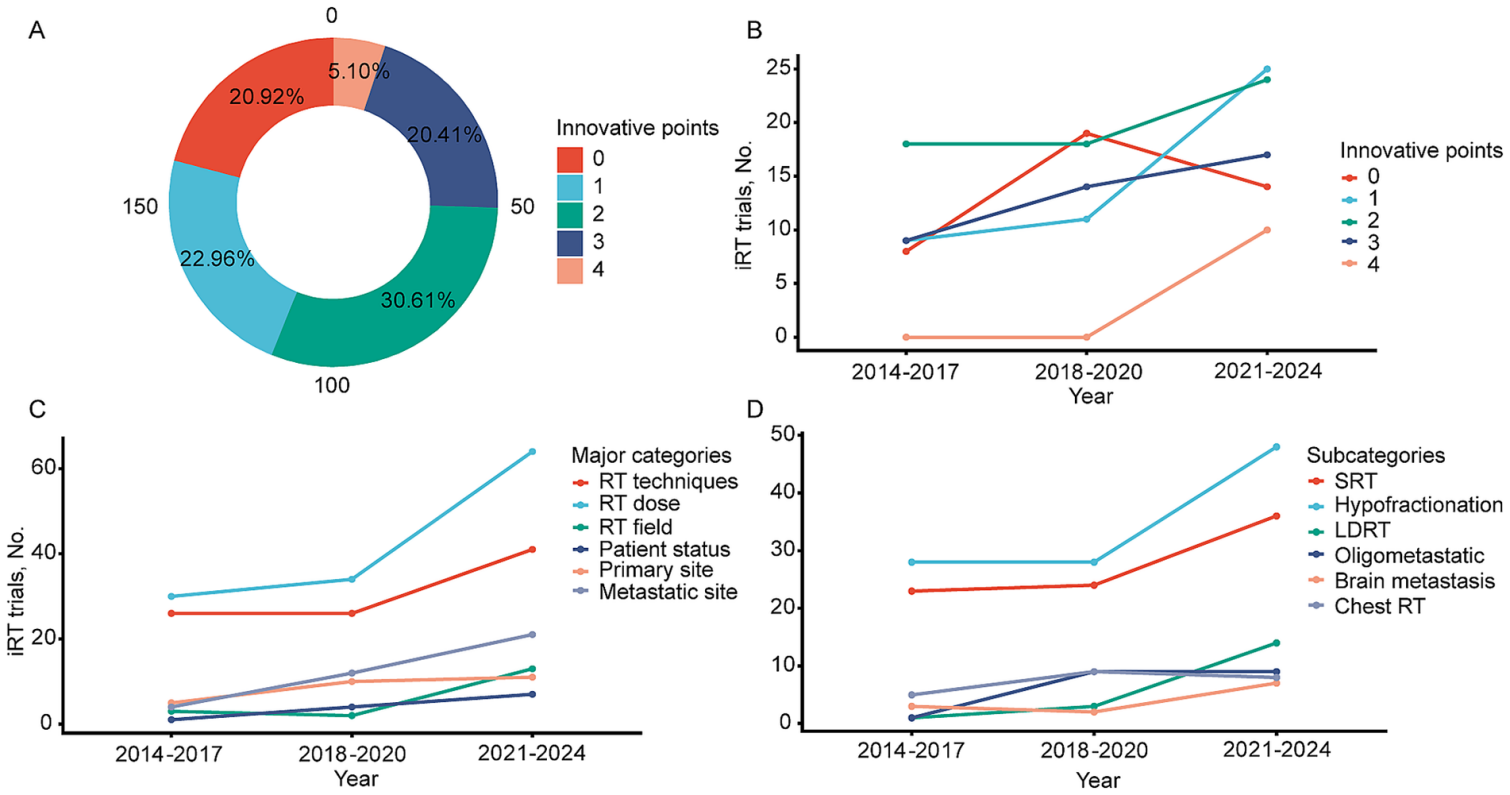
Changing trend of innovative points. **(A)** Proportion of research involved in different innovative points. **(B)** Changes in the number of studies related to different innovative points from 2014 to 2024. **(C)** Main categories of innovative points involve changes in the number of studies over time. **(D)** Subcategory of innovative points involves changes in the number of studies over time.

From 2014 to 2024, the number of innovative points per study ranged from 0 to 4 (major categories). Sixty trials (30.6%) had two innovative points, mainly on the optimization of radiotherapy technology and dose, with 43 trials specifically mentioning SRT. The remaining trials were divided into 0 (20.9%), 1 (23%), and 3 (20.4%) innovative points, with the least number (5.1%) having four innovative points (Figure 4A). Figure 4B shows the variation in the number of innovative points per trial over the years. From 2014 to 2024, the number of trials with zero innovative points initially rose and then declined, while trials with one to two innovative points rapidly increased. Trials with three innovative points showed a slow increase, and from 2021 to 2024, trials with four innovative points began to appear. To better illustrate the trend of specific innovative points, the number of trials with the 8 major categories and 36 subcategories of innovative points was summarized by time intervals (2014–2017, 2018–2020, and 2021–2024). Categories with ≤5 studies in any of the three periods were excluded, leaving 6 major categories and 6 subcategories. In 2014–2017, the focus of trials was primarily on SRT (23/44, 52.3%), with other innovative points scattered across different types. In 2018–2020, the focus remained largely on SRT (24/62, 38.7%), but the number of trials focusing on oligometastasis (9/62, 14.5%) and thoracic radiotherapy (9/62, 14.5%) increased. In 2021–2024, the focus on SRT continued (36/90, 40%), but there was a noticeable increase in the proportion of LDRT (14/90, 15.6%), as well as increased attention to radiation fields (13/90, 14.4%), patient status (7/90, 7.8%), and brain metastases (7/90, 7.8%) compared to previous periods (Figures 4C,D).

## Discussion

Many clinical studies have found that the synergistic effect between radiotherapy and ICIs is expected to change this situation. Welsh et al. (7) combined the analysis of PEMBRO-RT (phase 2) (8) and MDACC (phase 1/2) (9) clinical trials to avoid the problem of too small sample size in most studies and showed that radiotherapy was effective in increasing the sensitivity of advanced NSCLC to immunotherapy. However, there are still many questions to be solved about how this treatment mode of iRT is specifically applied to different patients. Clinical trials are essential for clinical practice and decision-making, and feature analysis of registered clinical trials can help us advance the iRT model. In recent years, the combination therapy model of iRT has been comprehensively studied, so we included 196 relevant clinical trials and statistically analyzed its research characteristics and innovations. It can be seen that with the development of time, researchers are continuously trying very different combination models and subdividing the patient population.

Thanks to the secondary analysis of the KEYNOTE-001 (10) study and the success of the PACIFIC (3) study, many investigators have started to focus on the iRT treatment paradigm, and the populations of these two studies are advanced and inoperable locally advanced NSCLC patients, respectively, and the study drugs are pembrolizumab and durvalumab, respectively. As can be seen, in this study, more than 31.1% of patients presented with Stage III NSCLC, including operable NSCLC; more than 39.8% of patients presented with Stage IV NSCLC, including oligometastases and brain metastases. With the deepening of the study, researchers have used iRT in early NSCLC, but it accounts for a relatively low proportion (more than 7.2%). Also deeply influenced by both studies, durvalumab was the most clearly mentioned drug in the included studies (30.1%), followed by pembrolizumab (15.8%), suggesting that durvalumab may be the most appropriate iRT modality.

In fact, the number of clinical trials focusing on radiotherapy is significantly lower than other oncology trials, with industry funding for radiotherapy clinical trials being relatively low (11). In this study, we observed that from 2014 to 2024, the number of iRT clinical trials has been steadily increasing, but 75.5% of these trials were led by hospitals and universities, with only 8.2% of the funding coming from the industry. This reflects a broader trend in radiotherapy research. While drug development is largely driven by pharmaceutical companies, advances in radiotherapy often originate from academic institutions or equipment manufacturers. These companies tend to focus on hardware innovation and rarely fund clinical trials, particularly those exploring biological mechanisms or combination therapies. One reason for this may be the challenges associated with commercializing radiotherapy technologies. Compared to pharmaceuticals, the market for new radiotherapy approaches is smaller, and the development cycle is longer, offering less immediate commercial return (12). As a result, there is limited industry interest in supporting radiotherapy trials, as well as those involving immunotherapy combinations. Addressing this gap may require alternative funding strategies, such as public–private partnerships or targeted support for technology-driven oncology research. This funding pattern may have several consequences. Trials without industry support often have limited budgets, making it harder to conduct large, multicenter studies. They may also progress more slowly and face a higher risk of early termination due to financial or operational constraints. In the long term, a lack of sustained funding may limit the ability to move from early-phase trials to larger confirmatory studies.

Another possible reason for the low proportion of industry-funded trials in this study is the limited range of immunotherapeutic agents included. To reduce complexity and focus on commonly studied combinations, we restricted the analysis to trials involving PD-1/PD-L1 and CTLA-4 inhibitors. As a result, some early-phase trials testing newer immunotherapy agents in combination with radiotherapy were not captured in our dataset. North America remains the leading region for radiotherapy clinical trials. In this study, 48.5% of trials were conducted in North America, followed by Asia (29.6%) and Europe (20.4%). Compared to data from Ma et al. (11), which reported 12% of radiotherapy trials in Asia between 2007 and 2017, the increase observed in our study suggests growing engagement from Asian institutions in iRT research in recent years.

High-level evidence sufficient to change guidelines for care is generally derived from large-sample, randomized controlled, multi-center, blinded, and data-monitoring committee phase 3 clinical studies (13). In our study, 56.1% of the trials were in phase 2, only 11.7% of the trials entered phase 3, and 55.6% of the trials had a sample size of less than 50, suggesting that the iRT field is still in the stage of active exploration. The number of single/multi-center trials is almost equally divided, and communication and cooperation among various academic groups are close, which is beneficial to the promotion of research and the generation of consensus. 36.7% of the trials used random allocation, twice as high as non-random allocation, compared with Huang et al. (14), who counted that only 27.6% of the randomized trials of SBRT clinical trials from 2010 to 2019, implying that researchers have paid more attention to the randomness of the trials in recent years, and then 48.5% of the trials did not label the allocation pattern, possibly due to 51.5% of the trials being single-arm intervention patterns. While 93.4% of trials were set to open-label, it may be because it is challenging to set blinding for iRT-type studies. 54.6% of the trials had a Data Monitoring Committee, which was lower than the historical data of 66.4% (11), suggesting that in the future researchers need to pay more attention to the Data Monitoring Committee to ensure the reliability of the study data (15).

Twenty-nine iRT clinical trials in NSCLC have been completed so far, but most of them are in phase 1–2, and the results are published in the form of papers and conference abstracts. Formenti et al. (16) used iRT to treat early lung cancer and randomly assigned 60 patients to durvalumab alone and duvalumab combined with SBRT, and major pathological responses (MPR) were observed in 16 patients in the combination group, compared with only 2 patients in the monotherapy group. Feigenberg et al. (17) used proton beam therapy (PBT) for reirradiation of recurrent thoracic lesions and consolidation therapy with pembrolizumab, with median progression-free survival (PFS) and overall survival (OS) of 8.8 and 22.8 months, respectively, and only one isolated in-field failure after reirradiation, but two patients developed grade 5 toxicity, so it may be necessary to carefully screen patients with thoracic recurrence of NSCLC for this therapy. Qiu et al. (18) used a split-course mode, from a fraction dose of 5 Gy to a total dose of 60 Gy, concurrent chemotherapy and ICI consolidation therapy, and a total of 18 patients were included, achieving a 100% objective response rate (objective response rate). Xue et al. (19) used LDRT combined with SBRT in the iRT modality for advanced treatment-naïve PD-L1-positive NSCLC, with a confirmed ORR (confirmed ORR) of 57.1% and a median PFS of 8.6 months, which was safe and tolerable.

25 (12.8%) studies have failed so far. Among these failed trials, slow enrollment (44%), safety issues (16%), and funding issues (12%) were the main reasons for failure, while in the failed randomized clinical trials in the field of radiation oncology from 2007 to 2010, Palma et al. (20) counted, the main reasons were from slow enrollment (57.5%) and lack of funding (15%), suggesting that researchers should pay more attention to the safety considerations of iRT when designing the protocol to avoid resulting in trial failure. However, regardless of the success or failure of the trial, these study designs are the path that researchers have explored, although the trial is stopped due to various reasons such as slow enrollment or funding, and can also enlighten us to design clinical trials in the future (21).

The innovation of clinical trials is not only reflected in the improvement of a certain treatment technology, but also involves the improvement of multiple aspects. Therefore, this study counts innovative points of various clinical studies for the convenience of statistics. It is important to clarify that an innovative point was included only if the research protocol explicitly mentioned a specific treatment method (e.g., SBRT), population (e.g., elderly), or site (e.g., brain metastasis). This approach is based on the assumption that if researchers prioritize a particular technique or population, they are likely to specify it clearly. In addition, from a contemporary perspective, 3D-CRT and IMRT are not regarded as an improvement of radiotherapy technology by researchers, and in fact, there are not many studies specifically labeling these two technologies, and most of them are vaguely processed as “radiotherapy.” In this study, 65.3% of the trials focused on radiotherapy dose optimization, and their exploration focused on dose hypofractionation. Corresponding to the improvement of radiotherapy techniques, mainly reflected in SRT, showing that SRT is the mainstream technology in iRT mode. Additionally, LDRT has shown a noticeable increase in recent years, accounting for 15.6% of dose optimization trials between 2021 and 2024. Unlike conventional radiotherapy, LDRT’s antitumor effect primarily stems from immunomodulatory mechanisms rather than direct cytotoxicity. LDRT can enhance systemic immune responses, including the abscopal effect, by promoting dendritic cell activation, improving antigen presentation, increasing T-cell infiltration, and inhibiting regulatory T cells (22). This growing interest reflects recognition of LDRT’s potential to improve outcomes in combination with immunotherapy. Regarding patient populations, 18.9% of trials focused on metastases, especially oligometastases and brain metastases. This indicates more refined application of iRT, where researchers aim to use aggressive local treatments to reduce tumor burden in NSCLC patients with specific metastatic states.

iRT trials mostly include 1–2 innovative points, matching common clinical trial designs. Over time, trials have shifted from vague populations and techniques to more detailed and diverse approaches. Future iRT trials are likely to explore more complex radiotherapy methods, such as dose painting to deliver varying doses within tumors, which may improve tumor control while sparing normal tissues. The integration of AI into radiotherapy planning and decision-making could enhance precision and individualization of treatment, potentially optimizing outcomes. Clinicians can expect more personalized treatment strategies based on patient characteristics and tumor biology, supported by evidence from increasingly sophisticated trials. For example, identifying which patients benefit most from irradiating lymph nodes or specific metastatic sites will improve therapeutic benefit and reduce unnecessary toxicity. For trial designers, these trends highlight the need to incorporate adaptive trial designs and biomarker-driven patient selection to better capture the heterogeneity of responses in iRT. Overall, these evolving trial designs and innovations are likely to accelerate the translation of iRT into clinical practice, enabling more effective and safer combination therapies. However, careful attention to trial quality, including adequate sample size, multicenter collaboration, and rigorous monitoring, remains essential to generate robust evidence that can guide treatment decisions.

This study also has several limitations. First, due to the limitations of the data source, not all relevant trials in this field may have been included. However, we believe the current dataset is sufficient to reflect overall trends. Second, the information on ClinicalTrials.gov is self-reported by investigators. Incomplete updates, irregular protocol descriptions, or missing details may lead to misclassification during data extraction and affect the accuracy of the results. Finally, our classification approach relies on explicit labeling in trial protocols. As conventional techniques such as IMRT were often not specified, this may have led to underestimation of technical diversity and introduced classification bias.

## Conclusion

The number of iRT clinical trials in NSCLC has continued to increase in recent years. It is mainly characterized by patients with stage III-IV NSCLC, and the study is mostly in phase 2, which is dominated by hospitals and universities. The representative drug is durvalumab. The investigators are more concerned about the optimization of radiotherapy dose and tend to use hypofractionation mode. Although the iRT model has been deeply studied, there are still many problems that have not been solved, and more precise and safe combined methods are expected to emerge in the future.

## Data Availability

The original contributions presented in the study are included in the article/Supplementary material, further inquiries can be directed to the corresponding author.
